# Diffuse Idiopathic Skeletal Hyperostosis Causing Progressive Dysphagia: A Case Report and Review

**DOI:** 10.1155/2023/8853575

**Published:** 2023-09-25

**Authors:** Farzin Davoodi, Narges Bazgir, Reza Naseri

**Affiliations:** ^1^Department of Otorhinolaryngology, Loghman Hakim Educational Hospital, Shahid Beheshti University of Medical Sciences, Tehran, Iran; ^2^Hearing Disorders Research Center, Shahid Beheshti University of Medical Sciences, Tehran, Iran; ^3^Department of Radiology, Loghman Hakim Educational Hospital, Shahid Beheshti University of Medical Sciences, Tehran, Iran

## Abstract

**Background:**

Diffuse idiopathic skeletal hyperostosis (DISH) is a rare noninflammatory disorder impacting spinal longitudinal ligament and enthesis. The majority of DISH cases are asymptomatic or have few manifestations. Manifestations include neck pain and stiffness, stridor, breathing disturbances, and dysphagia. *Case Presentation*. A mid-aged man with progressive dysphagia to solid food was admitted to Loghman Hakim Hospital. In cervical X-ray, a huge ossification in the anterior longitudinal ligament was evident. Eventually, he was diagnosed with DISH. Because of coronary artery disease, conservative treatment was considered for him.

**Conclusion:**

DISH is a rare disorder usually asymptomatic. In this case report, we present a DISH case with progressive dysphagia to solid foods.

## 1. Introduction

Diffuse idiopathic skeletal hyperostosis (DISH) also known as Forestier's disease was initially introduce in 1975 by Resnick. DISH is a noninflammatory disorder impacting spinal longitudinal ligament and enthesis. The affected regions gradually become ossified, thus lose the mobility [1–3]. DISH is a systemic disorder in which at least three continuous vertebras in anterolateral spine are ossified. The exact etiology of DISH remained to be cleared [1, 4]. The prevalence of DISH varies from 2.9 to 42% in articles [5, 6]. DISH is more frequent in males [2]. Majority of DISH cases are asymptomatic or have few manifestations. Manifestations include neck pain and stiffness, stridor, breathing disturbances, and dysphagia. Commonly, male cases are symptomatic [7]. Diabetes, metabolic syndrome, insulin-like growth factor, and obesity are among the factors that promote bone formation and make patients susceptible to DISH [8]. Simple X-ray imaging is usually enough to diagnose the DISH. Computed tomography (CT) scan and magnetic resonance imaging (MRI) are performed to evaluate the extent of the involvement [9, 10]. Lifestyle modification, steroid use, physiotherapy, and surgical resections are modalities to treat DISH [11, 12]. In this case report, we describe a mid-aged man who was admitted to Loghman Hakim Hospital due to dysphagia. He was eventually diagnosed with cervical DISH.

## 2. Case Presentation

**Figure 1:**
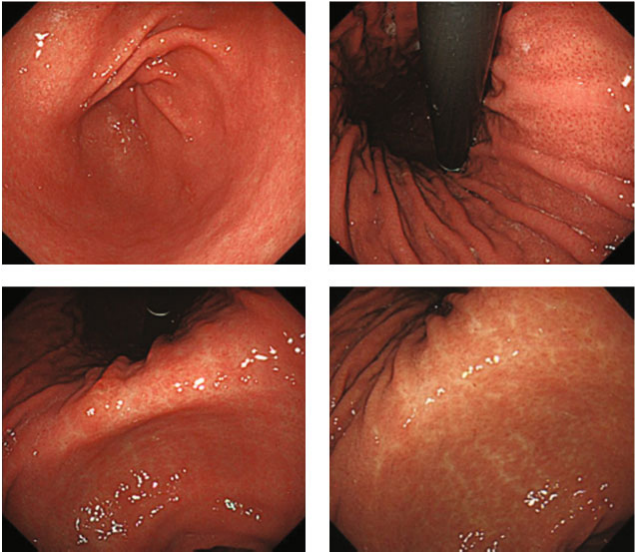
Endoscopy of the reported case.

A 72-year-old man with ischemic heart disease and insignificant surgical history was admitted to Loghman Hakim Hospital due to progressive dysphagia to solid foods. His dysphagia started about three months ago. He had complaint of cervical stiffness with more intensity in the mornings and severe cervical pain. On physical examination, no stiffness or limitation in mobility was significant. His neurological examinations were also normal. Because of progressive dysphagia to solid foods, he underwent endoscopy. No abnormality was observed on endoscopy (Figure 1).

**Figure 2:**
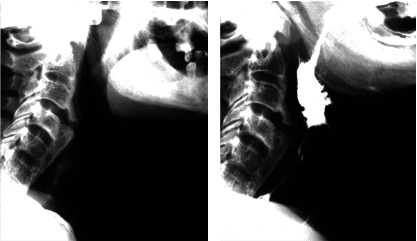
The barium swallow test of the patient.

Dysphagia was evident in barium swallow test.  Figure 2 illustrates the fluoroscopy.

**Figure 3:**
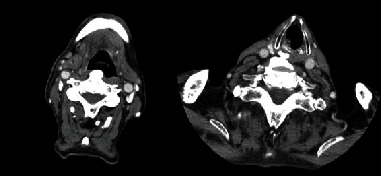
The axial CT scan of the patient's neck.

**Figure 4:**
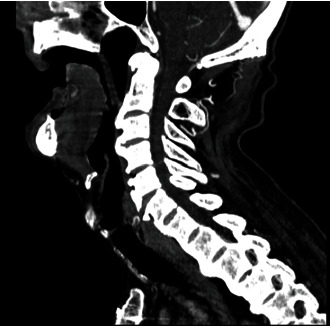
The sagittal CT scan of the patient's neck.

Cervical X-ray and computed tomography (CT) scan were performed. A huge ossification in anterior longitudinal ligament was in C2 to C4 level where a compressed esophagus was observed. No ossification in posterior longitudinal ligament was observed (Figures 3 and 4).

Because of ischemic heart disease with high risk to surgery in cardiology consult, the patient was not candidate for osteophyte removal. Thus, conservative treatments and physiotherapy were considered for him. Nonsteroidal anti-inflammatory drugs (NSAID) and physiology were prescribed for him. He was followed up for 24 months until his symptoms remitted subjectively. In the follow-ups, his dysphagia was remitted.

## 3. Discussion

DISH is a progressive noninflammatory disease involving entheses [2, 3]. The exact etiopathology of the disease is yet to be discovered. Genetic, metabolic, and vascular inflammatory factors are speculated to play a role in pathogenesis [13]. Any variation in Wnt signaling pathway may lead to changes in osteoblast activity and bone density [14]. Metabolic factors are composed of growth hormone, insulin-like growth factor, TGF-*β*1, and bone morphogenic protein 2 (BMP2) [11, 15]. Moreover, obesity is related to DISH formation. Higher body mass index (BMI) and waist circumference are associated with DISH [16]. Furthermore, diabetes is also correlated with DISH formation [17]. Despite of mentioning the inflammatory factors as possible cause in DISH, their role is not thoroughly confirmed [18]. The exact prevalence of DISH varies in different population, and it increases as people get older. In this article, we reported a mid-aged man with cervical DISH [2, 19]. DISH is less common in cervical than lumbar or thoracic region. The reported case also had cervical involvement [20]. Although mainly described as asymptomatic, DISH can cause serious manifestations such as dysphagia, shortness of breath, and airway obstruction [13]. DISH is currently diagnosed radiologically by three criteria: Resnick and Niwayama, Julkunen, and Utsinger [21–23]. The radiological evidence of DISH includes the presence of ossification in at least four contiguous vertebrae. It is important to rule out spondylarthritis and spondylosis [13]. For mild to moderate DISH, conservative treatments such as lifestyle modification, sedations, antireflux medications, nonsteroidal anti-inflammatory drug (NSAID), and muscle relaxants are considered. Corticosteroid injection is also a treatment modality. Surgical therapy is kept for those patients with progressive dysphagia and/or airway obstruction, those with no response to conservative treatments, or individuals with neurologic symptoms [11, 24, 25]. We reported a mid-aged man with progressive dysphagia to solid foods who was eventually diagnosed with DISH. Despite progressive dysphagia in the mentioned case, due to high cardiovascular risk, surgical removal was not considered for him. Overall, we reported a rare case with cervical DISH which causes progressive dysphagia to solid food. He was treated conservatively.

## Data Availability

The data of the case is available via inquiries from the corresponding author.
